# The relationship between non-suicidal self-injury and suicidal ideation in patients with borderline personality disorder treated at the Arrazi psychiatric hospital in Salé

**DOI:** 10.1192/j.eurpsy.2024.1357

**Published:** 2024-08-27

**Authors:** N. Ait Bensaid, F. El Omari

**Affiliations:** Arrazi psychiatric hospital in Salé, Salé, Morocco

## Abstract

**Introduction:**

Non-suicidal self-harm, i.e. the intentional self-infliction of bodily harm without apparent suicidal intent, is a powerful risk factor for suicidal ideation and behavior [1]. Although non-suicidal self-harm and suicidal behaviour are distinct concepts, the two forms of deliberate self-harm frequently coexist and share key instrumental functions, such as escaping aversive internal states, reducing dysphoria or communicating distress, especially in patients with personality disorders. [2]

Some individuals also report using non-suicidal self-harm to ameliorate suicidal thoughts or urges [2].

**Objectives:**

To assess the relationship between non-suicidal self-harm and suicidal ideation in patients with borderline personality disorder followed at the Arrazi psychiatric hospital in Salé.

**Methods:**

This was a descriptive cross-sectional study using a questionnaire including sociodemographic criteria, clinical criteria and the Beck suicidal intentionality scale to assess the relationship between non-suicidal self-harm and suicidal ideation in patients with borderline personality disorder followed and hospitalised at the Arrazi psychiatric hospital in Salé.

The inclusion criteria were as follows: both sexes with a diagnosis of borderline personality disorder according to DSM 5 criteria.

Exclusion criteria were current psychosis and severe intellectual disability.

**Results:**

We collect 63 participants.

The average age of the participants was 23, and they were predominantly female (89%). About 85% were single and 97% had no occupation. The majority of participants had a substance use disorder.

All participants had a history of non-suicidal self-harm and 36% had a history of suicide attempts.

Suicidal intent was strong in 45% of participants who had already attempted suicide.

Approximately 46% of participants reported that non-suicidal self-harm was intended to alleviate suicidal ideation and approximately 27% of participants reported having experienced suicidal ideation shortly after non-suicidal self-harm.

**Conclusions:**

Non-suicidal self-harm is very common in patients with borderline personality disorder often considered to have a mitigating effect on the internal stress of these patients and sometimes even neglected. The relationship between non-suicidal self-harm and suicidal ideation is an important one, and may reduce suicidal ideation in the short term but subsequently encourage further self-harm, thereby increasing the risk of suicide.

Particular attention must be paid to these patients and their self-harm, and specialised, comprehensive care is required.

**Disclosure of Interest:**

None Declared

